# Pericytes contribute to airway remodeling in a mouse model of chronic allergic asthma

**DOI:** 10.1152/ajplung.00286.2014

**Published:** 2015-01-30

**Authors:** Jill R. Johnson, Erika Folestad, Jessica E. Rowley, Elisa M. Noll, Simone A. Walker, Clare M. Lloyd, Sara M. Rankin, Kristian Pietras, Ulf Eriksson, Jonas Fuxe

**Affiliations:** ^1^Department of Medical Biochemistry and Biophysics, Matrix Division, Division of Vascular Biology, Karolinska Institutet, Stockholm, Sweden;; ^2^Leukocyte Biology Section, National Heart and Lung Institute, Sir Alexander Fleming Building, Imperial College London, London, United Kingdom; and; ^3^Lund University, Department of Laboratory Medicine Lund, Lund, Sweden

**Keywords:** asthma, house dust mite, airway hyperresponsiveness, pericyte, remodeling

## Abstract

Myofibroblast accumulation, subepithelial fibrosis, and vascular remodeling are complicating features of chronic asthma, but the mechanisms are not clear. Platelet-derived growth factors (PDGFs) regulate the fate and function of various mesenchymal cells and have been implicated as mediators of lung fibrosis. However, it is not known whether PDGF-BB signaling via PDGFRβ, which is critical for the recruitment of pericytes to blood vessels, plays a role in airway remodeling in chronic asthma. In the present study, we used a selective PDGFRβ inhibitor (CP-673451) to investigate the role of PDGFRβ signaling in the development of airway remodeling and lung dysfunction in an established mouse model of house dust mite-induced chronic allergic asthma. Unexpectedly, we found that pharmacological inhibition of PDGFRβ signaling in the context of chronic aeroallergen exposure led to exacerbated lung dysfunction and airway smooth muscle thickening. Further studies revealed that the inflammatory response to aeroallergen challenge in mice was associated with decreased PDGF-BB expression and the loss of pericytes from the airway microvasculature. In parallel, cells positive for pericyte markers accumulated in the subepithelial region of chronically inflamed airways. This process was exacerbated in animals treated with CP-673451. The results indicate that perturbed PDGF-BB/PDGFRβ signaling and pericyte accumulation in the airway wall may contribute to airway remodeling in chronic allergic asthma.

asthma is a heterogenic disease that currently affects 300 million people worldwide (34). Allergic asthma is defined as an allergen-induced, immune-driven chronic lung disease manifested by recurrent episodes of airway inflammation composed of infiltrating eosinophils, mast cells, macrophages, neutrophils, and lymphocytes (1). Airway remodeling is thought to be facilitated by this chronic inflammatory process (5). The structural changes associated with airway remodeling comprise goblet cell hyperplasia, collagen deposition, and increased airway smooth muscle (ASM) mass, all of which contribute to the clinical manifestation of lung dysfunction (1). Inflammatory cytokines and the profibrogenic growth factor transforming growth factor-β (TGF-β) are believed to play significant roles in driving these structural changes. However, the signaling mechanisms and the cellular sources from which the increased mesenchymal cell population of the airway wall is derived have not been fully described. Some studies have demonstrated that bone marrow-derived fibrocytes contribute to airway wall remodeling (30, 31), whereas others have suggested a minimal role for these cells (24). Additionally, the differentiation of mesenchymal cells from airway epithelial cells via epithelial-to-mesenchymal transition has been shown to be a mechanism of remodeling in a mouse model of severe allergic airway disease (20).

The platelet-derived growth factors (PDGFs) are mitogens for a number of mesenchymal cell types, including fibroblasts and smooth muscle cells (9). The receptors of the PDGFs, PDGF receptor alpha (PDGFRα) and PDGF receptor beta (PDGFRβ), are tyrosine kinases and are thus amenable to pharmacological intervention. However, little is known regarding the expression of the PDGFs in asthma, since the limited studies available in the literature have documented few differences in PDGF or PDGF receptor expression in human asthmatic patients compared with healthy controls (3, 6, 15, 25). Specifically focusing on PDGF-BB and its cognate receptor PDGFRβ in the context of allergic airway disease, stimulation of ASM cells with PDGF-BB in vitro has been shown to act in concert with TGF-β to stimulate cell migration (18). Moreover, a recent study using an adenovirus vector to overexpress PDGF-BB in the airway epithelium in an ovalbumin (OVA)-driven mouse model of asthma was shown to induce ASM cell proliferation and enhance airway hyperresponsiveness (AHR) (14). PDGF receptors are also expressed on vascular mural cells, a heterogeneous population of mesenchymal cells that line the outer surface of microvessels and are therefore abundant in the lung (2). Pericytes, the population of mural cells covering capillaries, express desmin and NG2 but are negative for α-smooth muscle actin (α-SMA), whereas mural cells covering arterioles and venules express desmin and α-SMA and are termed vascular smooth muscle (VSM) cells (2). Mural cells are recruited to and retained on blood vessels through PDGF-BB/PDGFRβ interactions (reviewed in Ref. 2). Impaired pericyte coverage of blood vessels is seen in PDGF-BB-deficient mice and in diseases like cancer and is associated with vascular leakage and edema (2, 4, 32).

In light of these findings, and since tyrosine kinase inhibitors such as masitinib are currently being investigated as asthma therapies (16), we elected to investigate the impact of PDGFRβ inhibition on airway and VSM cells/pericytes in a mouse model of chronic aeroallergen exposure driven by exposure to house dust mite (HDM) extract via the respiratory mucosa. HDM exposure is strongly associated with human asthma and is one of the most ubiquitous respiratory allergens worldwide. In mice, chronic respiratory HDM exposure leads to Th2-polarized airway inflammation, remodeling of the airway wall and bronchial hyperreactivity, and thus recapitulates many of the features of clinical asthma (21). Using this paradigm, we investigated the role of PDGFRβ signaling and the downstream effects of inhibiting this receptor during chronic HDM exposure on airway remodeling and lung dysfunction.

## MATERIALS AND METHODS

### 

#### Animal handling.

Female C57Bl/6 mice were bred in-house at the Karolinska Institutet animal facility at the Department of Mikrobiologi, Tumör- och Cellbiologi or purchased from Harlan Laboratories (Wyton, UK) and housed at the central animal facility at Imperial College London. Transgenic mice used for pericyte lineage tracing studies [Tg(Cspg4-DsRed.T1)1Akik/J] were obtained from the Jackson Laboratories (Bar Harbor, ME); the phenotype of these mice was determined by direct fluorescent imaging of the DsRed fluorescent signal in ear biopsies. Animals were initiated into experiments at 8–12 wk of age. Mice were housed under specific pathogen-free conditions following a 12-h light-dark cycle and were provided food and water ad libitum. All experiments described in this study were approved by the Research Ethics Committees at the Karolinska Institute and at Imperial College London and were performed in accordance with the UK Home Office and Imperial College London guidelines on animal welfare.

#### Antigen and drug administration.

Mice were challenged with purified *Dermatophagoides pteronyssinus* extract (HDM) (Greer Laboratories, Lenoir, NC) 5 days/wk for 3 or 5 consecutive wk. HDM was resuspended in sterile phosphate-buffered saline (PBS) (2.5 mg of protein/ml); 10 μl of HDM were administered intranasally to isoflurane-anesthetized mice (Sigma-Aldrich, Gillingham, UK) (21). Control mice were subjected to the same exposure protocol but received PBS (10 μl). In each group, a subset of animals received a selective PDGFRβ inhibitor [1-(2-(5-(2-methoxy-ethoxy)-benzoimidazol-1-yl)-quinolin-8-yl)-piperdin-4-ylamine (CP-673451); Pfizer, New York, NY] by oral gavage [33 mg/kg in polyethyleneglycol 400 (PEG-400); Sigma-Aldrich] daily for a period of 5 consecutive wk. Other animals received the drug vehicle (PEG-400). CP-673451 has 10-fold higher affinity for PDGFRβ vs. PDGFRα and greater than 450-fold higher affinity for PDGFRβ vs. c-Kit and was prepared according to the procedures described previously (27).

#### Lung function measurements.

After 5 wk of allergen and drug administration, mice were subjected to lung function measurements (8). Mice were injected intraperitoneally with pentobarbital sodium (50 mg/kg) (Sigma-Aldrich), followed by an intramuscular injection of ketamine (90 mg/kg) (Sigma-Aldrich) (29). Mice were tracheostomized with a 19-gauge blunt-ended needle. Measurements of dynamic AHR and elastance were acquired by use of the flexiVent system (Scireq, Montréal, Canada). Mice were ventilated, and airway resistance and elastance were calculated as previously described (29). A 4- to 6-μm nebulizer was used, and the duration of nebulization was 10 s per dose. A 50% duty cycle was used during nebulization. Fluctuations in AHR and elastance were determined by the responses of the total respiratory system to increasing concentrations (10–300 mg/ml) of methacholine (MCh) (Sigma-Aldrich) delivered into the inspiratory line of the flexiVent ventilator. The P_ao_ (pressure at airway opening) waveform was monitored throughout flexiVent data acquisition and was found to be similar during measurements as when the initial calibration was performed before running each subject. Measurements were not taken when there were obvious signs of respiratory effort. Following flexiVent data acquisition, the thoracic cavity of each mouse was opened to confirm that cardiac arrest had not occurred.

#### Collection of specimens.

Mice were humanely euthanized and the lungs were dissected. In some experiments, bronchoalveolar lavage (BAL) was collected (0.45 ml of PBS). BAL cells were counted by use of a hemocytometer, centrifuged at 1,200 rpm for 5 min, then mounted on glass slides by use of a Cytospin (Thermo Scientific, Hemel Hempstead, UK) and stained with hematoxylin and eosin for differential cell counts. BAL supernatants were stored at −20°C, then submitted to ELISA to determine mouse PDGF-BB expression according to the manufacturer's instructions (R&D Systems, Abingdon, UK). In other experiments, mice were perfused through the left ventricle with 1% paraformaldehyde, and the trachea, extrapulmonary bronchi, and lung were dissected out. The tracheas, extrapulmonary bronchi, and lungs were isolated and preserved in 1% paraformaldehyde for 30 min, then transferred to PBS. The lung was embedded in Optimal Cutting Temperature compound, stored at −80°C, and cut into 10-μm-thick sections for staining. Fresh lung samples were snap-frozen for quantitative PCR (qPCR) and immunoblotting experiments.

#### Immunoblotting.

Frozen lung tissue specimens were homogenized in 400 μl of lysis buffer, which was composed of T-PER Tissue Protein Extraction Reagent (Rockford, IL) supplemented with protease inhibitors (Complete Mini tablets from Roche Diagnostics Scandinavia, Bromma, Sweden), and homogenized with a homogenizer (Percell, Stockholm, Sweden). Protein samples were separated by SDS-PAGE on 4–12% polyacrylamide gels and blotted onto nitrocellulose membranes. The membranes were incubated in blocking buffer (5% skim milk) in Tris-buffered saline containing 0.5% Tween for 1 h at room temperature and probed with a polyclonal rabbit anti-human PDGF-BB antibody (Aviva Systems Biology, San Diego, CA) diluted 1:1,000 in blocking buffer. After washing, the membranes were incubated with a horseradish peroxidase-conjugated secondary anti-rabbit antibody (GE Healthcare, Stockholm, Sweden) diluted 1:5,000 in blocking buffer, for 1 h at room temperature. The membranes were developed using ECL+ detection reagent (Amersham, GE Healthcare). To ascertain equal loading of protein, the membranes were stripped and reprobed with a rabbit anti-mouse antibody against Akt (Cell Signaling/BioNordika Sweden, Stockholm, Sweden) diluted 1:1,000 in blocking buffer.

#### Immunofluorescent staining.

Lung sections from C57Bl6 and DsRed-NG2 mice were warmed to room temperature and nonspecific binding was blocked by incubating in 5% normal goat serum (NGS), 0.3% Triton X-100 and 0.1% bovine serum albumin (BSA) (all purchased from Sigma-Aldrich) in PBS for 1 h at room temperature. Sections were stained using the appropriate primary (overnight) and secondary (1 h) antibodies (Table 1). After being washed twice in PBS, sections were mounted by use of a glycerol-based mounting medium containing DAPI (Vector Laboratories, Peterborough, UK) to visualize DNA. For polyclonal antibodies, negative reagent controls were carried out.

**Table 1.** Antibodies used to stain tracheobronchal whole mounts and lung sections

Application	Antibody	Supplier	Catalog Number	Concentration
Immunofluorescence: Lung sections				
Primary antibodies	Rabbit anti-mouse NG2	Millipore	AB5320	1:200
	Cy3-conjugated anti-α-smooth muscle actin	Sigma-Aldrich	C6198	1:1,000
	FITC-conjugated anti-α-smooth muscle actin	Sigma-Aldrich	F3777	1:500
	Armenian hamster anti-mouse CD31	Abcam	ab78739	1:500
Immunofluorescence: Tracheas				
Primary antibodies	Rabbit anti-mouse NG2	Millipore	AB5320	1:500
	Cy3-conjugated anti-α-smooth muscle actin	Sigma-Aldrich	C6198	1:1,000
	Rabbit anti-mouse desmin	Abcam	ab6570	1:400
	Rat anti-mouse anti-E-cadherin	Thermo Scientific/Pierce	MA1-25160	1:500
	Armenian hamster anti-mouse CD31	Abcam	ab78739	1:400
Immunofluorescence				
Secondary antibodies	Alexa Fluor 488-conjugated 2° antibody goat anti-rat	Jackson ImmunoResearch	111-545-143	1:500
	Alexa Fluor 488-conjugated 2° antibody goat anti-rabbit	Jackson ImmunoResearch	111-545-144	1:500
	Alexa Fluor 546-conjugated 2° antibody goat anti-rabbit	Invitrogen	A-11035	1:500
	Alexa Fluor 647-conjugated 2° antibody goat anti-Armenian hamster	Jackson ImmunoResearch	127-605-099	1:500

For tracheobronchial whole-mount immunostaining, the trachea and extrapulmonary bronchi were dissected and cleaned of connective tissue and mounted on Kwikgard-coated (WPI, Sarasota, FL) six-well culture plates. Nonspecific binding was blocked by incubating trachea sections in 5% NGS, 0.3% Triton X-100, and 0.2% BSA in PBS overnight. Left and right trachea sections were stained using the appropriate primary and secondary antibodies overnight at room temperature (Table 1). Sections were washed thrice in PBS and mounted using a glycerol-based mounting medium containing DAPI (Vector Laboratories). For all antibodies, negative reagent controls were carried out with no staining observed.

#### Light microscopy.

Quantification of α-SMA- and NG2-positive cells in the trachea and extrapulmonary bronchi was performed with a Leica DM2500 fluorescence microscope (Leica Microsystems, Milton Keynes, UK). Counts were normalized to the length of each tracheobronchial whole mount (including the extrapulmonary bronchi at the distal end of the preparation). For image acquisition from immunostained tracheobronchial whole mounts and lung sections, 1,024 × 1,024 pixel RGB-color images were obtained by using a Zeiss LSM 510 inverted confocal microscope with argon, helium-neon, and UV lasers (Carl Zeiss, Göttingen, Germany) using an EC Plan-Neofluar ×40/1.30 oil objective with the DIC phase contrast technique (10). Using the optimized pinhole size, 0.9-μm-thick optical sections were sequentially collected with a step size of 1 μm (lung sections) or 1.5 μm (tracheas). Image analysis was performed by use of Zeiss AIM 3.2.2 software and Zeiss LSM Image software (Carl Zeiss AG). Three-dimensional visualizations (Supplemental Videos S1–S4; Supplemental Material for this article is available on the Journal website) of confocal images were prepared with Volocity software (Perkin-Elmer, Waltham, MA). Morphometric analysis of ASM thickness was performed by a blinded observer on α-SMA-stained lung sections and analyzed with ImageJ software (NIH). Morphometric analysis of the number of NG2^+^ cells in the airway subepithelial region was conducted by a blinded observer on lung sections stained for NG2, α-SMA, and CD31 (to prevent enumeration of pericytes associated with the microvasculature). Five representative images were taken of the large airways (bronchi and bronchioles) from each animal (*n* = 5–10 per group from two independent experiments). Images were taken on a Zeiss LSM 510 inverted confocal microscope and processed by using ImageJ. NG2^+^ cells in the airway subepithelial region were enumerated and counts were normalized to millimeters of basement membrane.

#### qPCR.

Whole tissue RNA from lung was isolated with TRIzol reagent (Invitrogen/Life Technologies, Stockholm, Sweden) and QIAGEN RNeasy (Qiagen, Sollentuna, Sweden) according to the manufacturers' instructions. Total RNA (1 μg) was reverse transcribed according to the manufacturer's instructions (iScript cDNA synthesis kit, Bio-Rad). qPCR was performed by using Platinum SYBR green SuperMix (Invitrogen) and 25 ng cDNA per reaction. Primers used were Pdgfb QT00266910, Pdgfrb QT00113148, and L19 QT01779218. Ct values for the three genes analyzed were 19–20 (PDGF-BB), 24–26 (PDGFRβ), and 17–18 (L19). Expression levels were normalized to the expression of L19. Melt curves were assessed following the completion of RT-PCR to ensure that the fluorescent signal resulted from a single PCR product.

#### Data analysis.

Data were analyzed with GraphPad Prism Software version 6 (GraphPad Software, San Diego, CA). Statistical analysis was performed by Student's *t*-test or one-way ANOVA with a subsequent Tukey's post hoc test where appropriate. Differences were considered statistically significant when *P* < 0.05. Pearson correlation analysis was performed to assess the relationship between the degree of ASM thickening and numbers of cells. Results are presented as means ± SE.

## RESULTS

### 

#### PDGFRβ blockade exacerbates airway hyperresponsiveness driven by chronic HDM exposure.

**Fig. 1.**
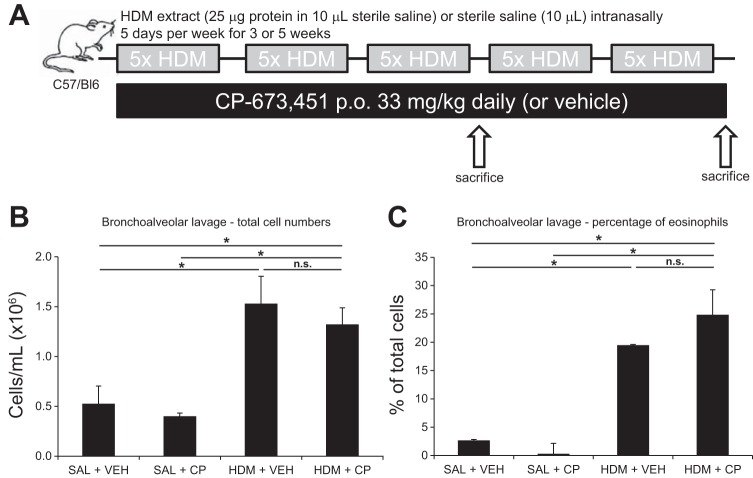
*A*: schedule of allergen dosing and drug delivery in the house dust mite (HDM) model. Bronchoalveolar lavage (BAL) was collected after 3 wk of HDM exposure to assess the severity of inflammation (*B*) and the proportion of eosinophils (*C*) recruited to the airways of saline (SAL) and HDM-exposed mice [intranasally (i.n.) 5 days/wk for 3 wk] treated daily with a PDGFRβ inhibitor (CP-673451; CP) or vehicle (PEG-400; VEH); *n* = 5 mice per group. *P < 0.05; n.s., not significant.

To evaluate the impact of impaired PDGFRβ signaling on allergic inflammation, we treated mice with the tyrosine kinase inhibitor CP-673451, which selectively inhibits PDGFRβ (27), in the context of HDM exposure for a period of 3 consecutive wk (Fig. 1*A*). Systemic administration of CP-673451 did not have any effect on the degree of airway inflammation or the proportion of eosinophils in the BAL of mice exposed to saline or HDM for 3 wk (Fig. 1, *B* and *C*). To determine the physiological impact of respiratory HDM exposure and systemic CP-673451 treatment, lung function was assessed in mice exposed to saline or HDM for 5 wk (±CP-673451) in response to aerosolized MCh.

**Fig. 2.**
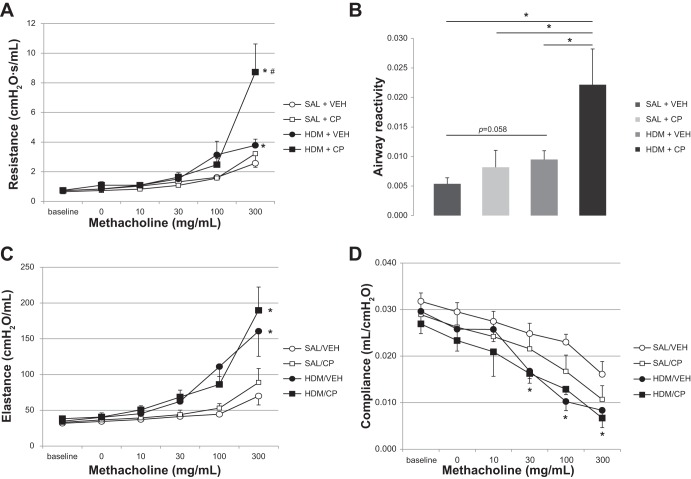
Airway hyperreactivity was assessed by using the flexiVent system in mice exposed to either sterile saline or HDM (25 μg) intranasally for 5 wk (5 days/wk) and treated daily with a PDGFRβ inhibitor (CP-673451) or vehicle (PEG-400). Airway hyperresponsiveness to increasing doses of aerosolized methacholine (*A*), airway reactivity (the slope of the dose-response curve) (*B*), airway elastance (*C*), and airway compliance (*D*) were measured. *P < 0.05 vs. SAL+VEH, #*P* = 0.05 vs. HDM+VEH; *n* = 4–6 mice per group.

Airway resistance and elastance were measured in control animals (PBS+VEH; PBS+CP), in HDM-exposed mice (HDM+VEH), as well as in HDM-exposed mice treated with CP-673451 (HDM+CP). CP-673451 had no significant effect on lung function in the saline control groups (SAL+VEH and SAL+CP) (Fig. 2, *A*–*D*). Relative to control mice, HDM+VEH animals demonstrated significantly higher airway resistance (*P* < 0.05), and, compared with HDM+VEH mice, HDM+CP-exposed animals demonstrated significantly increased airway resistance (*P* < 0.05) (Fig. 2*A*). Airway reactivity [calculated as the slope of the MCh dose-response curve from baseline to the peak of the dose-response curve, as previously described (23)] showed an increased trend in the HDM group relative to control (*P* = 0.05) but was significantly increased in HDM+CP-exposed animals (*P* < 0.05) (Fig. 2*B*). Maximal airway elastance was significantly greater in HDM-exposed mice vs. the control animals (*P* < 0.05) (Fig. 2*C*); conversely, airway compliance was significantly lower in the HDM groups vs. the saline control groups (*P* < 0.05) (Fig. 2*D*). No differences in airway elastance or compliance were found between the HDM+VEH and HDM+CP groups (Fig. 2, *C* and *D*).

#### PDGFRβ blockade during HDM exposure promotes ASM mass thickening.

**Fig. 3.**
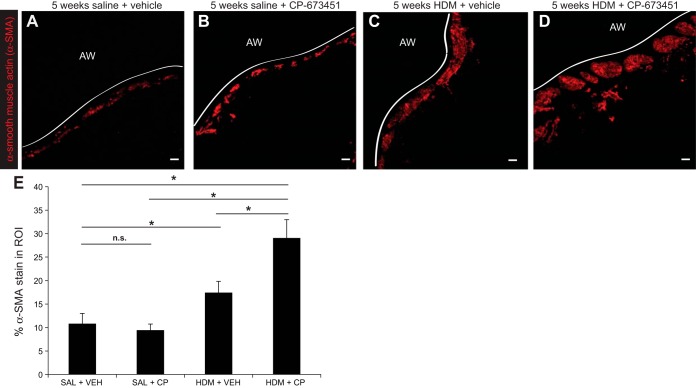
*A*–*D*: immunostaining of lung sections was performed with an α-smooth muscle actin antibody (α-SMA, red) to assess smooth muscle mass in the region of interest (ROI; 40 μm distal to the subepithelial basement membrane) of the large airways (AW) in saline and HDM-exposed mice (i.n. 5 days/wk for 5 wk) treated daily with a PDGFRβ inhibitor (CP-673451) or vehicle (PEG-400). Solid white line indicates the subepithelial basement membrane. Scale bar 10 μm. *E*: morphometric analysis of α-SMA staining in the airways of saline and HDM-exposed mice (i.n. 5 days/wk for 5 wk) treated daily with a PDGFRβ inhibitor (CP-673451) or vehicle (PEG-400). *P < 0.05, *n* = 5–7 from 2 independent experiments.

The lung function results suggested that inhibition of PDGFRβ signaling exacerbated disease in HDM-exposed mice, so we performed a morphometric analysis of ASM mass (Fig. 3, *A*–*D*). Quantification of the α-SMA^+^ stained area in the subepithelial region of the large airways revealed a significant increase in ASM mass in HDM-VEH mice, which was increased further in HDM-CP mice (Fig. 3*E*).

#### Loss of vascular smooth muscle cells upon HDM exposure.

**Fig. 4.**
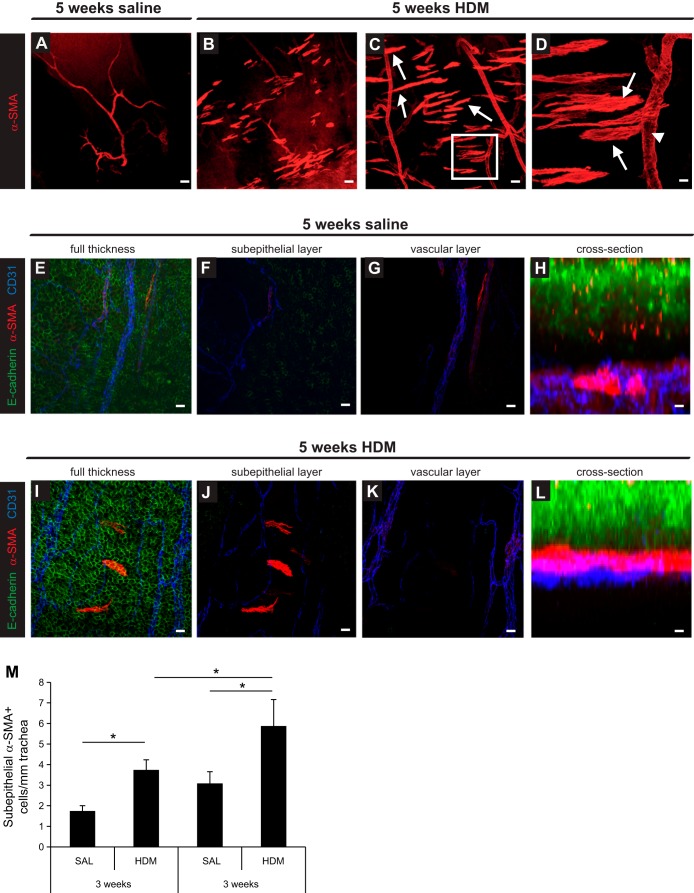
*A*–*L*: whole-mount immunostaining for α-SMA (red) and the microvasculature in the extrapulmonary bronchus (CD31, blue) in relation to the airway epithelium (E-cadherin, green) was performed to determine the location α-SMA^+^ cells in the bronchus in saline and HDM-exposed mice. Arrows (*C* and *D*) indicate subepithelial α-SMA^+^ cells and arrowheads indicate gaps in the pericyte coverage of vessels. *H* and *L*: Z-stack confocal imaging was performed to assess the 3-dimensional location of α-SMA^+^ cells in the bronchus in saline- and HDM-exposed mice. *M*: quantification of subepithelial α-SMA^+^ cells in the bronchus after 3 and 5 wk of saline or HDM exposure. *P < 0.05; *n* = 5–10 mice per group from 3 independent experiments. Scale bars 50 μm (*A* and *B*), 25 μm (*C*, *E*–*G*, *I*–*K*), and 12.5 μm (*D*, *H*, *L*).

Since PDGFRβ signaling plays a vital role in the recruitment of mural cells to blood vessels we next examined whether mural cell coverage of airway blood vessels was affected during HDM-induced airway inflammation. We performed whole-mount staining of extrapulmonary bronchi with markers against CD31, α-SMA, and E-cadherin to visualize endothelial cells, vascular smooth muscle cells, and epithelial cells, respectively; negative control whole mounts (omission of the primary antibodies) were negative for all markers (data not shown). Confocal microscopy analysis of stained specimens revealed marked changes in the association of α-SMA^+^ VSM cells with airway blood vessels in HDM-exposed animals. Under noninflammatory conditions, α-SMA^+^ VSM cells were tightly associated with arterioles and venules in the mouse airways (Fig. 4*A*, Supplemental Video S1). Following HDM exposure, α-SMA^+^ cells that were not associated with vessels were identified in the mouse airways (Fig. 4, *B*–*D*). High-magnification imaging analysis indicated the loss of α-SMA^+^ VSM cells from airway blood vessels (Fig. 4*D*, Supplemental Video S2). Further studies demonstrated tight association of VSM cells with the microvasculature in control mice and the presence of α-SMA^+^ cells close to the airway subepithelium in HDM-exposed mice (Fig. 4, *E*–*L*). Quantification showed a significant increase in the number of subepithelial α-SMA^+^ positive cells in mice exposed to HDM for 3 wk, which increased further following 5 wk of HDM exposure (Fig. 4*M*).

#### Loss of NG2^+^ pericytes upon HDM exposure.

**Fig. 5.**
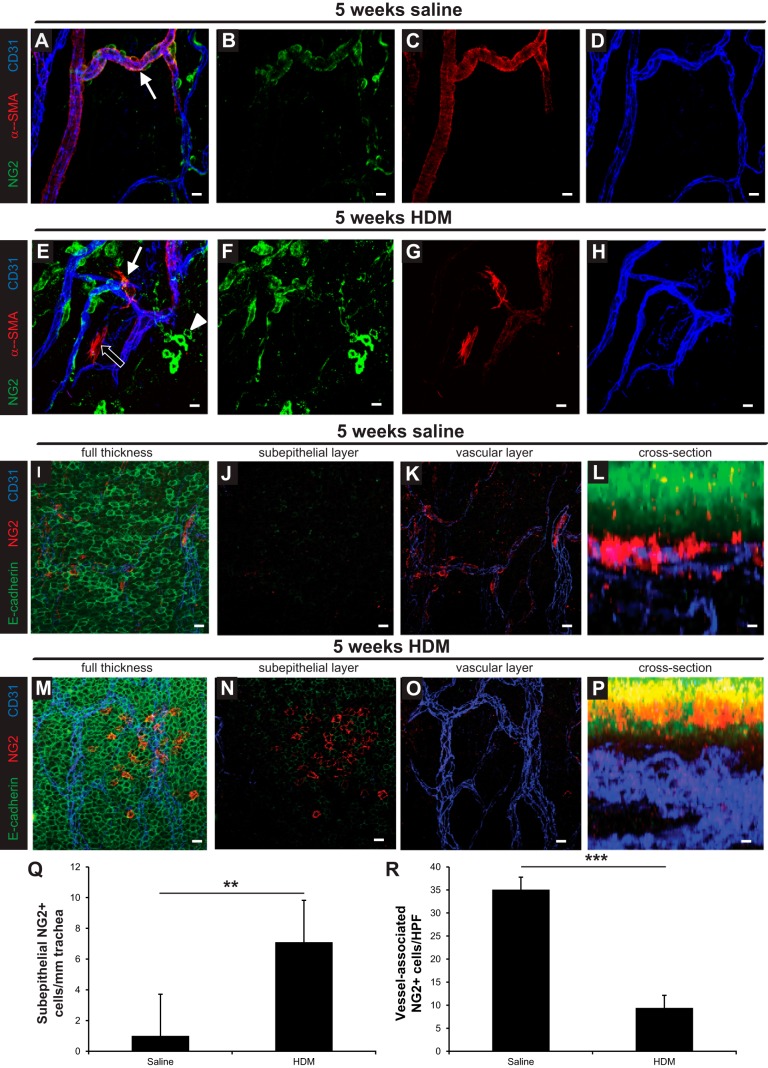
*A*–*H*: immunostaining of the trachea and extrapulmonary bronchus for NG2 (green), α-SMA (red), and CD31 (blue) with Z-stack confocal imaging was performed to assess the 3-dimensional location of pericytes and the distribution of pericyte marker expression in saline and HDM-exposed mice (i.n. 5 days/wk for 5 wk). Scale bars 25 μm. *I*–*P*: immunostaining of the trachea and extrapulmonary bronchus for E-cadherin (green), NG2 (red), and CD31 (blue) with Z-stack confocal imaging was performed to assess the 3-dimensional location of pericytes in the bronchus of saline and HDM-exposed mice (i.n. 5 days/wk for 5 wk). Scale bars 25 μm (*I*–*K*, *M*–*O*) and 12.5 μm (*L* and *P*). Quantification of subepithelial (*Q*) and vessel-associated (*R*) NG2^+^ cells in the bronchus after 5 wk of saline or HDM exposure. **P < 0.01, ***P < 0.001; *n* = 10 mice per group from 2 independent experiments.

Next, we stained whole-mount preparations of extrapulmonary bronchi for the pericyte marker NG2 as well as α-SMA to characterize this cell population under normal conditions (Fig. 5, *A*–*D*) and following HDM exposure (Fig. 5, *E*–*H*); negative control whole mounts (omission of the primary antibodies) were negative for all markers (data not shown). Confocal imaging revealed that pericytes associated with the airway microvasculature were α-SMA^+^/NG2^+^ (Fig. 5*A*, white arrow), α-SMA^+^/NG2^−^ (Fig. 5*E*, black arrow), or α-SMA^−^/NG2^+^ (Fig. 5*E*, white arrowhead); all populations of pericytes appeared to be more loosely associated with the airway microvasculature following 5 wk of HDM exposure (Fig. 5*E*). Further experiments showed that NG2^+^ pericytes were tightly associated with airway capillaries under baseline conditions (Fig. 5, *I*–*L*, Supplemental Video S3). Following 5 wk of HDM exposure, NG2^+^ cells appeared to be lost from the microvasculature (Fig. 5, *M*–*P*, Supplemental Video S4). In parallel, NG2^+^ cells were detected below the airway epithelium (Fig. 5*N*). Quantification confirmed that the number of vessel-associated NG2^+^ cells was decreased while the number of subepithelial NG2^+^ cells was increased in the extrapulmonary bronchi in HDM-exposed mice vs. control mice (Fig. 5, *Q* and *R*).

#### Accumulation of NG2^+^ cells in the airway wall upon HDM exposure.

**Fig. 6.**
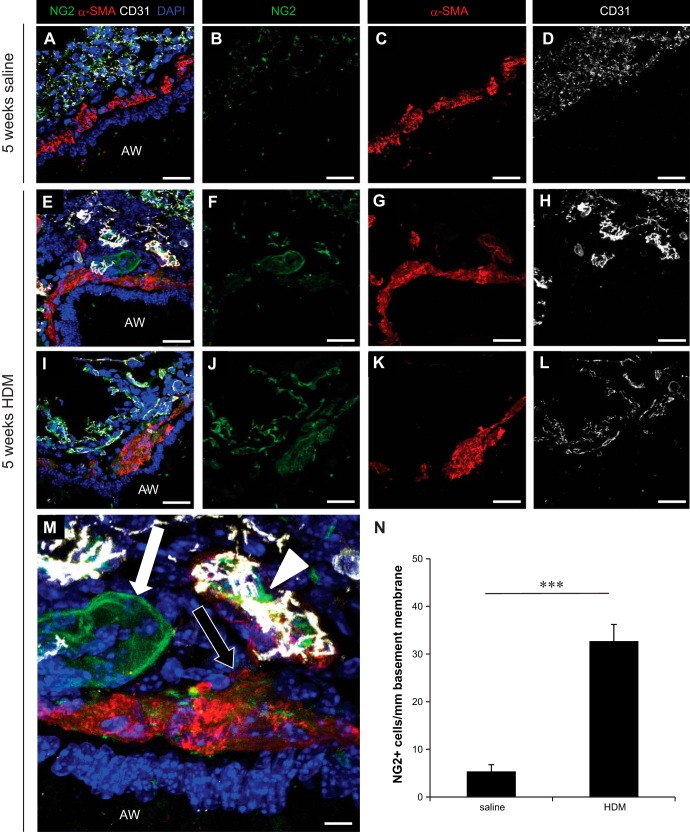
*A*–*L*: immunostaining of lung sections for NG2 (green), α-SMA (red), CD31 (white), and cell nuclei (DAPI; blue) was performed to assess pericyte localization in the wall of large airways in saline and HDM-exposed mice (i.n. 5 days/wk for 5 wk). Scale bars 25 μm (*A*–*L*) and 100 μm (*M*). White arrow indicates NG2^+^ cells present in the subepithelial region of the airway wall, black arrow indicates NG2^+^/α-SMA^+^ cells present in the subepithelial region of the airway wall, and arrowhead indicates microvessel-associated NG2^+^ cells (*M*). *N*: morphometric quantification of the number of NG2^+^ cells present in the subepithelial region of the airway wall in control mice (saline) and mice exposed to HDM for 5 wk (HDM). ***P < 0.001; *n* = 5–10 mice from 2 independent experiments.

To further assess the localization of pericytes in the airways and how it was affected by HDM exposure, NG2, α-SMA, and CD31 immunostaining was performed on frozen lung sections; negative control lung sections (omission of the primary antibodies) were negative for all markers (data not shown). In control animals, pericytes were localized exclusively around pulmonary blood vessels and associated with alveolar capillaries (Fig. 6, *A*–*D*). In mice exposed to HDM (Fig. 6, *E*–*L*), NG2^+^ cells were found associated with microvessels (indicated by arrowheads; Fig. 6*M*). Moreover, NG2^+^/α-SMA^+^ cells (indicated by black arrows) and NG2^+^ cells (indicated by white arrows; Fig. 6*M*) were found in the airway subepithelial region, associated with smooth muscle bundles surrounding the large airways. Morphometric analysis of five representative images from saline- and HDM-exposed mice revealed a statistically significant increase in the number of NG2^+^ cells that were not associated with the microvasculature in the airway wall following 5 wk of HDM exposure (Fig. 6*N*, *P* < 0.001).

**Fig. 7.**
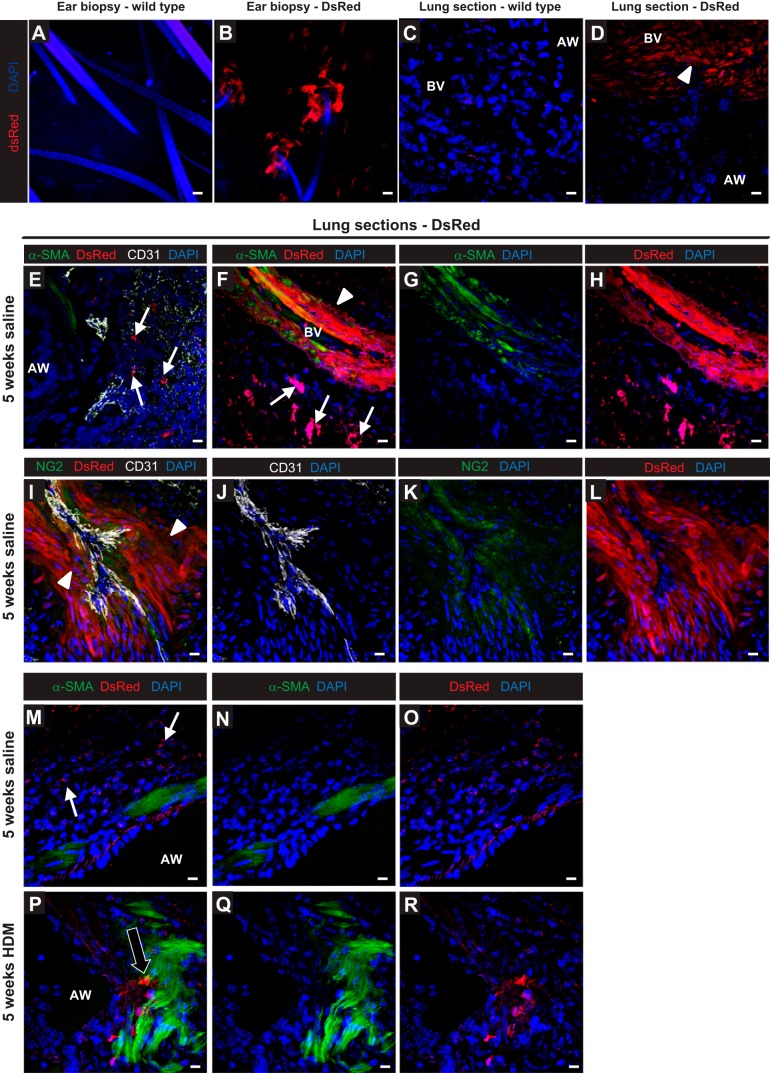
Pericyte localization was assessed in ear biopsies (*A* and *B*) and in the lungs (*C* and *D*) of wild-type and Tg(Cspg4-DsRed.T1)1Akik/J saline control mice by direct imaging of the DsRed fluorescent signal. The identity of DsRed-positive cells in the lungs was assessed by colocalization of the signal in α-SMA^+^ (*E*–*H*) and NG2-expressing (*I*–*L*) pericytes in the pulmonary blood vessels (BV). Immunostaining of lung sections from DsRed-NG2 transgenic mice for α-SMA (green), DsRedNG2^+^ pericytes (red), CD31 (white), and cell nuclei (DAPI; blue) was performed in saline control (*M*–*O*) and HDM-exposed (*P*–*R*) mice (i.n. 5 days/wk for 5 wk). White arrows indicate DsRed-NG2^+^ cells present in the lung parenchyma (*E*, *F*, *M*), black arrow indicates DsRed-NG2^+^/α-SMA^+^ cells present in the airway smooth muscle (ASM) bundles (*P*), and arrowheads indicate microvessel-associated DsRedNG2^+^ cells (*F*, *I*). Scale bars 25 μm (*A*–*R*).

To further study the effect of HDM on pericyte localization in the mouse airways, we used transgenic NG2DsRedBAC reporter mice [Tg(Cspg4-DsRed.T1)1Akik/J]. These mice express the red fluorescent protein DsRed.T1 under the control of the mouse NG2 (Cspg4) promoter and were thus used to interrogate whether pericytes accumulate in the ASM following chronic HDM exposure (Fig. 7). The genotype of the mice was confirmed by observation of ear biopsies under a fluorescent microscope; bright red NG2^+^ cells were observable surrounding the base of hair follicles in transgene-bearing mice (Fig. 7, *A* and *B*). We were able to observe DsRed-NG2 cells in the walls of large vessels (Fig. 7, *D*–*H*), and DsRed-NGS cells were also positive for NG2 by antibody-mediated staining (Fig. 7, *I*–*L*). DsRed-NG2 cells were absent from the ASM bundles of saline control mice (Fig. 7, *M*–*O*), whereas clusters of DsRed-NG2 cells were clearly visible within the ASM bundles of mice exposed to HDM for 5 wk (Fig. 7, *P*–*R*).

#### Reduced lung PDGF-BB expression and PDGFRβ signaling in HDM mice.

**Fig. 8.**
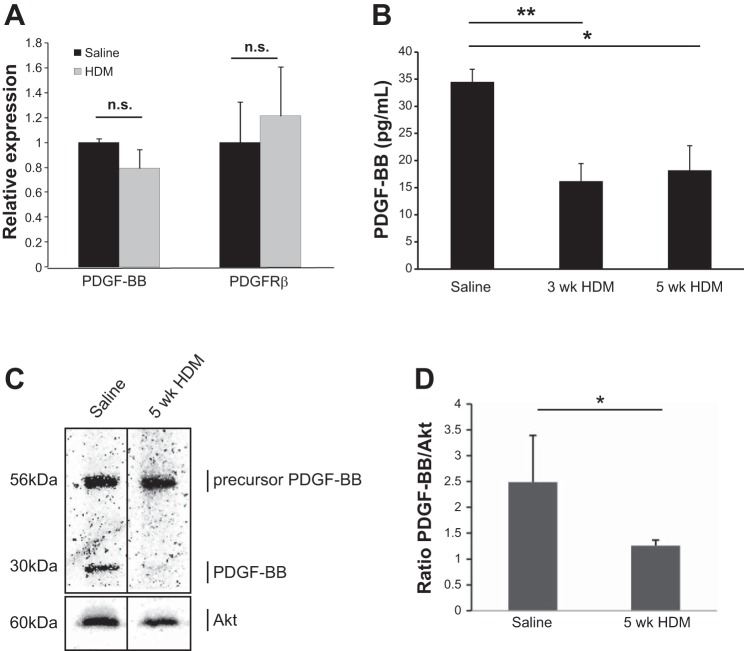
*A*: qPCR analysis of PDGF-BB and PDGFRβ mRNA expression in whole-lung lysates from saline and HDM-exposed mice (i.n. 5 days/wk for 5 wk); *n* = 5 representative of 2 independent experiments. ELISA analysis of PDGF-BB expression in BAL supernatants (*B*) and immunoblotting for PDGF-BB protein expression (*C*) in whole-lung lysates from saline and HDM-exposed mice (i.n. 5 days/wk for 5 wk); *n* = 5–6 (ELISA) and *n* = 3 (immunoblotting) per group, representative of 2 independent experiments. The immunoblot shown in *C* presents representative lanes for saline and HDM-exposed mice from 3 replicates per group run on the same gel. *D*: quantification of PDGF-BB expression assessed by immunoblotting in whole lung homogenates from saline and HDM-exposed mice. *P < 0.05, **P < 0.01.

We next assessed the expression of PDGF-BB and PDGFRβ in the lungs of mice exposed to saline or HDM on the mRNA and protein levels. qPCR analysis of PDGF-BB and PDGFRβ expression in the lung revealed no changes in the expression of these genes after 5 wk of HDM compared with saline mice (Fig. 8*A*). ELISA performed on BAL supernatants revealed significantly less PDGF-BB expression in the airways of mice exposed to HDM for 3 or 5 wk compared with control mice (Fig. 8*B*). Immunoblotting analysis showed unaltered levels of the PDGF-BB precursor in lungs from HDM mice vs. saline mice (Fig. 8*C*). However, a significant decrease in the cleaved, active form of PDGF-BB was found in HDM-exposed mice (Fig. 8*D*). In line with this, we found that PDGFRβ signaling was reduced in the lungs of mice exposed to HDM for 3 wk and severely compromised after 5 wk (Fig. 8*E*).

#### PDGFRβ blockade exacerbates pericyte loss from blood vessels.

**Fig. 9.**
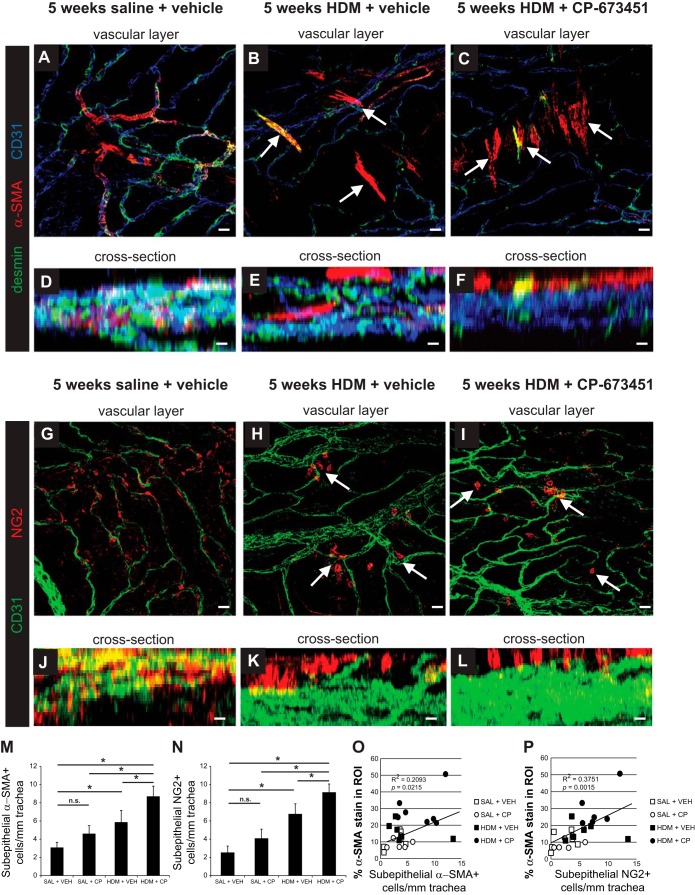
Whole-mount trachea and extrapulmonary bronchus immunostaining for α-SMA (red), desmin (green), and CD31 (blue) (*A*–*F*) or NG2 (red) and CD31 (green) (*G*–*L*) was performed with Z-stack confocal imaging (*D*–*F*, *J*–*L*) to determine the location of pericytes in the bronchus of saline and HDM-exposed mice (i.n. 5 days/wk for 5 wk) treated daily with a PDGFRβ inhibitor (CP-673451) or vehicle (PEG-400). Quantification and Pearson correlation with ASM thickness of subepithelial α-SMA^+^ cells (*M* and *N*) and NG2^+^ cells (*O* and *P*) in the bronchus after 5 wk of saline or HDM exposure in mice treated daily with a PDGFRβ inhibitor (CP-673451) or vehicle (PEG-400). White arrows indicate pericytes that have detached from blood vessels. *P < 0.05; *n* = 5–7 mice per group from 2 independent experiments.

Finally, we wanted to determine whether pericyte loss from airway blood vessels was exacerbated in mice exposed to HDM+CP mice vs. HDM-VEH. Confocal microscopy analysis of whole-mount preparations of extrapulmonary bronchi indicated that fewer α-SMA^+^ (Fig. 9, *A*–*F*) and NG2^+^ (Fig. 9, *G*–*L*) cells were associated with the airway microvasculature in mice exposed to HDM+CP mice vs. HDM-VEH mice. Quantification verified an increase of subepithelial α-SMA^+^ and NG2^+^ cells in HDM-CP mice vs. HDM-VEH mice (Fig. 9, *M* and *O*). No effect on pericyte dissociation from blood vessels was seen in saline-exposed mice treated daily with CP-673451 vs. mice given the vehicle only. Pearson correlation analysis demonstrated a moderate but significant correlation between the numbers of subepithelial α-SMA^+^ VSM cells (Fig. 9*N*) and NG2^+^ cells pericytes (Fig. 9*P*) and the degree of ASM thickening.

## DISCUSSION

Chronic allergic asthma is a complex disease provoked by aeroallergen exposure, which promotes Th2-polarized airway inflammation (13) and airway remodeling (1), for which a number of possible underlying mechanisms have been proposed (7, 12, 19, 20, 22, 26, 30). We aimed to investigate the impact of PDGFRβ inhibition on HDM-induced allergic airway disease in mice. HDM exposure resulted in increased airway resistance, increased elastance, and reduced compliance (Fig. 2), in agreement with previous studies showing that airway inflammation and remodeling contribute to AHR (21). However, HDM-exposed animals treated with CP-673451 presented worse lung dysfunction, with increased maximal respiratory resistance and airway reactivity compared with HDM-exposed mice given the vehicle (Fig. 2, *A* and *B*). Clear differences in AHR were observed between the HDM-exposed groups, suggesting that impaired PDGFRβ signaling promotes lung dysfunction under chronic inflammatory conditions. To investigate this, we evaluated ASM thickening in these animals. Rather than the expected reduction in ASM thickening, since PDGF-BB is known to be a smooth muscle mitogen (9), we instead observed increased ASM thickening in mice exposed to HDM and treated with the PDGFR inhibitor (Fig. 3).

Since PDGFRβ signaling is critical for endothelial-pericyte cell interactions, we studied whether airway microvessel-associated pericytes may have contributed to airway remodeling by being recruited to the airway wall. These experiments showed the loss of α-SMA^+^ cells from the microvasculature of the bronchi and accumulation of these cells in the subepithelial region of the airway wall (Fig. 4). To confirm that these cells were indeed pericytes and not tissue fibroblasts acquiring a myofibroblast phenotype, we assessed the localization of NG2^+^ pericytes and observed the accumulation of the cells in the subepithelial region of the large airways as well, similar to what was observed with α-SMA^+^ pericytes (Fig. 5). Moreover, NG2^+^ pericytes appeared to be incorporated into ASM bundles in mice exposed to HDM (Fig. 6). Lineage tracing experiments with DsRed-NG2 mice, in which pericytes expressing NG2 exhibit a red fluorescent marker, confirmed the presence of pericytes within the ASM bundles of mice exposed to HDM (Fig. 7). These results suggest that pericytes may represent a source of airway-resident mesenchymal cells that can contribute to airway remodeling in chronic asthma. However, an alternative process could also be considered to explain these findings, since a change in the phenotype in airway wall resident mesenchymal cells (i.e., fibroblasts) may have occurred with the acquisition of pericyte markers such as NG2.

Although the expression levels of PDGF-BB mRNA and pro-PDGF-BB were not significantly altered in HDM-exposed mice compared with control mice, the amount of processed, active PDGF-BB was reduced in the lungs of HDM exposed mice (Fig. 8), providing a mechanism to explain the observed change in pericyte location. This suggests that the activity of one or more pro-PDGF-BB convertases may have been inhibited during the allergic inflammatory response driven by HDM exposure. Several proteinases have been reported to cleave pro-PDGF-BB into its active form and it would be interesting in the future to study their role in regulating PDGF-BB signaling and pericyte biology in allergic asthma (33).

In support of reduced PDGF-B/PDGFRβ signaling as a mechanism promoting pericyte loss from the microvasculature and airway remodeling, we found increased pericyte loss in the extrapulmonary bronchi when HDM-exposed mice were treated daily with CP-673451 (Fig. 9). These results resemble data from a recent study using a model of airway inflammation in the mouse evoked by inoculation with *Mycoplasma pulmonis* bacteria. In that study, loss of pericyte coverage from airway blood vessels was observed during the first week after infection and was associated with reduced expression of PDGF-BB (10). Pharmacological inhibition of PDGF-BB with a selective PDGF-BB aptamer exaggerated pericyte loss and led to more severe *Mycoplasma pulmonis* infection.

Our results demonstrate that PDGF-B/PDGFRβ signaling is important not only for recruitment of pericytes to blood vessels during development, but also to maintain pericyte attachment to blood vessels in adult tissues. Reduced PDGF-B/PDGFRβ signaling in inflamed tissues might weaken endothelial cell-pericyte interactions and, subsequently, promote pericyte loss from blood vessels. It remains to be determined which driving forces attract pericytes toward inflamed airways. A number of cytokines and chemokines are overexpressed in asthmatic airways and are under investigation in this model.

Recently, pericytes were identified as a source of myofibroblasts in a mouse model of inflammatory kidney fibrosis (17, 24), during scar formation in a mouse model of spinal cord injury (11), as well as in a mouse model of bleomycin-induced lung fibrosis (28). The results of this study provide further evidence of a role for pericytes in fibrotic lung disease. Taken together, these findings led us to propose a role for pericyte accumulation in the airway wall, where pericytes are incorporated into the ASM and thereby contribute to AHR in response to chronic respiratory HDM exposure. The identification of lung-resident perivascular cell accumulation in the airway wall provides evidence for a novel mechanism of airway remodeling and ultimately contributes to our knowledge of the pathology of chronic allergic asthma. However, elucidation of the mechanism by which this occurs is at an early stage, and further studies are needed to determine the chemotactic signals driving pericyte accumulation in the airway wall, as well as the ability of these cells to differentiate into a more smooth muscle cell-like phenotype.

## GRANTS

This work was supported by research grants from the Swedish Research Council, the Swedish Childhood Cancer Foundation, the Swedish Cancer Society, and the Strategic Cancer Foundation (StratCan) at Karolinska Institute to J. Fuxe, and by research grants from the Swedish Wenner-Gren Foundation, the Karolinska Institute, Imperial College London and the UK Medical Research Council to J. R. Johnson.

## DISCLOSURES

No conflicts of interest, financial or otherwise, are declared by the author(s).

## AUTHOR CONTRIBUTIONS

J.R.J., E.F., K.P., U.P.-E.E., and J.F. conception and design of research; J.R.J., E.F., J.E.R., E.M.N., and S.A.W. performed experiments; J.R.J., E.F., J.E.R., and E.M.N. analyzed data; J.R.J., E.F., C.M.L., S.M.R., K.P., U.P.-E.E., and J.F. interpreted results of experiments; J.R.J., E.F., and J.E.R. prepared figures; J.R.J. and J.F. drafted manuscript; J.R.J., C.M.L., S.M.R., and J.F. edited and revised manuscript; J.R.J., E.F., J.E.R., E.M.N., S.A.W., C.M.L., S.M.R., K.P., U.P.-E.E., and J.F. approved final version of manuscript.
